# Diaqua­bis(4-formyl­benzoato-κ*O*)zinc(II)

**DOI:** 10.1107/S1600536808003152

**Published:** 2008-02-06

**Authors:** Zhao-Peng Deng, Shan Gao, Li-Hua Huo, Seik Weng Ng

**Affiliations:** a School of Chemistry and Materials Science, Heilongjiang University, Harbin 150080, People’s Republic of China; b Department of Chemistry, University of Malaya, 50603 Kuala Lumpur, Malaysia

## Abstract

The Zn^II^ atom in the title compound, [Zn(C_8_H_5_O_3_)_2_(H_2_O)_2_], which lies on a twofold rotation axis, is coordinated by two monodentate carboxyl­ate groups and two water mol­ecules in a tetra­hedral geometry; the geometry is distorted towards octa­hedral owing to two long Zn⋯O_carbon­yl_ contacts [2.512 (2) Å]. Hydrogen-bonding inter­actions give rise to a three-dimensional network. The formyl group is disordered approximately equally over two positions.

## Related literature

A pseudo-polymorph of the title compound containing a solvent water mol­ecule exists in a *P*2/*c* modification, which features zinc in an unambiguous tetra­hedral coordination geometry; see Deng *et al.* (2006 ▶).
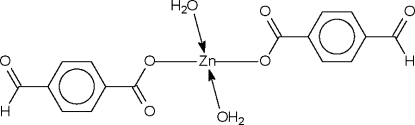


## Experimental

### 

#### Crystal data

[Zn(C_8_H_5_O_3_)_2_(H_2_O)_2_]
*M* *_r_* = 399.64
Monoclinic, 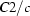

*a* = 27.537 (1) Å
*b* = 5.0039 (2) Å
*c* = 12.0930 (6) Å
β = 110.039 (2)°
*V* = 1565.4 (1) Å^3^
*Z* = 4
Mo *K*α radiation
μ = 1.61 mm^−1^
*T* = 295 (2) K
0.34 × 0.26 × 0.18 mm

#### Data collection

Rigaku R-AXIS RAPID diffractometer
Absorption correction: multi-scan (*ABSCOR*; Higashi, 1995 ▶) *T*_min_ = 0.532, *T*_max_ = 0.760
7203 measured reflections
1785 independent reflections
1448 reflections with *I* > 2σ(*I*)
*R*_int_ = 0.034

#### Refinement

*R*[*F* ^2^ > 2σ(*F* ^2^)] = 0.029
*wR*(*F* ^2^) = 0.082
*S* = 1.09
1785 reflections
132 parameters
3 restraints
H atoms treated by a mixture of independent and constrained refinement
Δρ_max_ = 0.59 e Å^−3^
Δρ_min_ = −0.38 e Å^−3^

### 

Data collection: *RAPID-AUTO* (Rigaku, 1998 ▶); cell refinement: *RAPID-AUTO*; data reduction: *CrystalStructure* (Rigaku/MSC, 2002 ▶); program(s) used to solve structure: *SHELXS97* (Sheldrick, 2008 ▶); program(s) used to refine structure: *SHELXL97* (Sheldrick, 2008 ▶); molecular graphics: *X-SEED* (Barbour, 2001 ▶); software used to prepare material for publication: *publCIF* (Westrip, 2008 ▶).

## Supplementary Material

Crystal structure: contains datablocks global, I. DOI: 10.1107/S1600536808003152/bt2669sup1.cif

Structure factors: contains datablocks I. DOI: 10.1107/S1600536808003152/bt2669Isup2.hkl

Additional supplementary materials:  crystallographic information; 3D view; checkCIF report

## Figures and Tables

**Table 1:** Selected bond lengths (Å)

Zn1—O1*W*	1.983 (2)
Zn1—O1	2.005 (2)
Zn1—O2	2.512 (2)

**Table 2:** Hydrogen-bond geometry (Å, °)

*D*—H⋯*A*	*D*—H	H⋯*A*	*D*⋯*A*	*D*—H⋯*A*
O1*W*—H1*W*1⋯O1^i^	0.84 (1)	1.93 (1)	2.761 (2)	174 (3)
O1*W*—H1*W*2⋯O2^ii^	0.84 (1)	1.88 (1)	2.720 (2)	174 (3)

Symmetry codes: (i) 
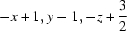
; (ii) 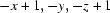.

**Fig. 1.**
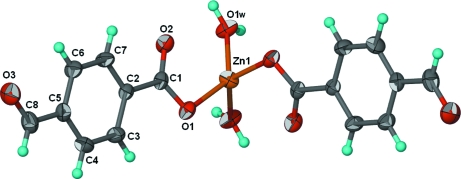
Anisotropic displacement parameter plot of (I). Displacement ellipsoids are drawn at the 50% probability level and H atoms as spheres of arbitrary radius.

## supplementary crystallographic information

### Comment

A pseuodpolymorph of the title compound containing a solvent water molecule was isolated from the reaction of zinc acetate and 4-formylbenzoic acid in the presence of sodium hydroxide (Deng *et al.*, 2006). The reaction with pyridine in place of sodium hydroxide yielded the title polymorph.

### Experimental

Zinc diacetate dihydrate (2.2 g, 10 mmol) was added to anaqueous solution of 4-formylbenzoic acid (3.0 g, 20 mmol) that has earlier been treated with 1 ml pyridine to give a pH of 6. The solution was allowed to evaporate at room temperature; colorless prismatic crystals separated from the filtered solution after several days. C&N elemental analysis. Calc. C_16_H_14_O_8_Zn: C 48.08, H3.53%. Found: C 48.06, H 3.56%.

### Refinement

The formyl group is disordered over two sites; the ratio of the site occupation factors refined to a 0.508 (5):0.492 (5) ratio.

The carbon-bound H atoms were placed in calculated positions [C–H 0.93 Å and *U*_iso_(H) 1.2*U*_eq_(C)], and were included in the refinement in the riding-model approximation. The water H-atoms were located in a difference Fourier map, and were refined with distance restraints of O–H 0.85±0.01 Å and H···H 1.39±0.01 Å; their displacement parameters were freely refined.

### Crystal data

**Table:**

[Zn(C_8_H_5_O_3_)_2_(H_2_O)_2_]	*F*_000_ = 816
*M**_r_* = 399.64	*D*_x_ = 1.696 Mg m^−^^3^
Monoclinic, *C*2/*c*	Mo *K*α radiation λ = 0.71073 Å
Hall symbol: -C 2yc	Cell parameters from 5776 reflections
*a* = 27.537 (1) Å	θ = 3.2–27.5º
*b* = 5.0039 (2) Å	µ = 1.61 mm^−^^1^
*c* = 12.0930 (6) Å	*T* = 295 (2) K
β = 110.039 (2)º	Block, colorless
*V* = 1565.4 (1) Å^3^	0.34 × 0.26 × 0.18 mm
*Z* = 4	

### Data collection

**Table:**

Rigaku R-AXIS RAPID diffractometer	1785 independent reflections
Radiation source: fine-focus sealed tube	1448 reflections with *I* > 2σ(*I*)
Monochromator: graphite	*R*_int_ = 0.034
Detector resolution: 10.000 pixels mm^-1^	θ_max_ = 27.5º
*T* = 295(2) K	θ_min_ = 3.2º
ω scans	*h* = −35→35
Absorption correction: multi-scan(ABSCOR; Higashi, 1995)	*k* = −6→6
*T*_min_ = 0.532, *T*_max_ = 0.760	*l* = −15→15
7203 measured reflections	

### Refinement

**Table:**

Refinement on *F*^2^	Secondary atom site location: difference Fourier map
Least-squares matrix: full	Hydrogen site location: inferred from neighbouring sites
*R*[*F*^2^ > 2σ(*F*^2^)] = 0.029	H atoms treated by a mixture of independent and constrained refinement
*wR*(*F*^2^) = 0.082	*w* = 1/[σ^2^(*F*_o_^2^) + (0.0444*P*)^2^ + 0.5739*P*] where *P* = (*F*_o_^2^ + 2*F*_c_^2^)/3
*S* = 1.09	(Δ/σ)_max_ = 0.001
1785 reflections	Δρ_max_ = 0.59 e Å^−^^3^
132 parameters	Δρ_min_ = −0.38 e Å^−^^3^
3 restraints	Extinction correction: none
Primary atom site location: structure-invariant direct methods	

### Fractional atomic coordinates and isotropic or equivalent isotropic displacement parameters (Å^2^)

**Table:**

	*x*	*y*	*z*	*U*_iso_*/*U*_eq_	Occ. (<1)
Zn1	0.5000	0.07300 (7)	0.7500	0.03500 (15)	
O1W	0.52887 (7)	−0.2045 (3)	0.67306 (15)	0.0420 (4)	
O1	0.44080 (6)	0.3312 (3)	0.69827 (13)	0.0347 (4)	
O2	0.46297 (6)	0.2522 (3)	0.54394 (14)	0.0396 (4)	
O3	0.28940 (13)	1.2782 (7)	0.2481 (3)	0.0537 (13)	0.508 (5)
O3'	0.25060 (16)	1.2320 (10)	0.3707 (4)	0.0757 (18)	0.492 (5)
H1W1	0.5398 (10)	−0.346 (3)	0.7104 (19)	0.051 (8)*	
H1W2	0.5313 (11)	−0.208 (5)	0.6055 (12)	0.062 (9)*	
C1	0.43534 (8)	0.3714 (4)	0.59025 (19)	0.0299 (5)	
C2	0.39595 (8)	0.5736 (4)	0.52428 (18)	0.0283 (4)	
C3	0.35743 (9)	0.6552 (5)	0.5675 (2)	0.0396 (5)	
H3	0.3554	0.5809	0.6363	0.048*	
C4	0.32199 (9)	0.8489 (6)	0.5070 (2)	0.0444 (6)	
H4	0.2963	0.9046	0.5357	0.053*	
C5	0.32469 (8)	0.9588 (4)	0.4047 (2)	0.0348 (5)	
C6	0.36283 (9)	0.8759 (5)	0.3612 (2)	0.0373 (5)	
H6	0.3645	0.9485	0.2919	0.045*	
C7	0.39830 (9)	0.6846 (5)	0.4219 (2)	0.0358 (5)	
H7	0.4240	0.6303	0.3932	0.043*	
C8	0.28767 (9)	1.1697 (5)	0.3399 (2)	0.0460 (6)	
H8	0.2621	1.2233	0.3691	0.055*	0.508 (5)
H8'	0.2924	1.2551	0.2760	0.055*	0.492 (5)

### Atomic displacement parameters (Å^2^)

**Table:**

	*U*^11^	*U*^22^	*U*^33^	*U*^12^	*U*^13^	*U*^23^
Zn1	0.0384 (2)	0.02089 (19)	0.0487 (3)	0.000	0.01878 (17)	0.000
O1W	0.0676 (11)	0.0278 (8)	0.0359 (10)	0.0119 (8)	0.0247 (8)	0.0052 (7)
O1	0.0445 (9)	0.0313 (8)	0.0266 (8)	−0.0005 (7)	0.0101 (6)	0.0054 (6)
O2	0.0460 (9)	0.0351 (8)	0.0355 (9)	0.0121 (7)	0.0111 (7)	−0.0003 (7)
O3	0.051 (2)	0.055 (2)	0.052 (2)	0.0156 (17)	0.0141 (17)	0.0215 (19)
O3'	0.058 (3)	0.086 (4)	0.079 (3)	0.036 (2)	0.019 (2)	0.008 (3)
C1	0.0335 (11)	0.0244 (10)	0.0276 (11)	−0.0051 (8)	0.0051 (8)	−0.0011 (8)
C2	0.0318 (10)	0.0251 (10)	0.0264 (10)	−0.0022 (8)	0.0078 (8)	−0.0022 (9)
C3	0.0413 (12)	0.0478 (13)	0.0338 (13)	0.0033 (11)	0.0180 (10)	0.0055 (10)
C4	0.0356 (12)	0.0557 (15)	0.0458 (15)	0.0093 (11)	0.0190 (10)	−0.0025 (12)
C5	0.0314 (11)	0.0341 (12)	0.0337 (12)	0.0016 (9)	0.0044 (9)	−0.0032 (10)
C6	0.0418 (12)	0.0386 (12)	0.0322 (12)	0.0061 (10)	0.0135 (9)	0.0093 (10)
C7	0.0417 (12)	0.0366 (11)	0.0342 (12)	0.0106 (10)	0.0196 (10)	0.0037 (10)
C8	0.0368 (13)	0.0432 (13)	0.0497 (16)	0.0091 (11)	0.0043 (11)	−0.0036 (12)

### Geometric parameters (Å, °)

**Table:**

Zn1—O1w	1.983 (2)	C2—C3	1.395 (3)
Zn1—O1w^i^	1.983 (2)	C3—C4	1.392 (4)
Zn1—O1^i^	2.005 (2)	C3—H3	0.9300
Zn1—O1	2.005 (2)	C4—C5	1.379 (4)
Zn1—O2	2.512 (2)	C4—H4	0.9300
O1W—H1W1	0.84 (1)	C5—C6	1.389 (3)
O1W—H1W2	0.84 (1)	C5—C8	1.490 (3)
O1—C1	1.279 (3)	C6—C7	1.385 (3)
O2—C1	1.241 (3)	C6—H6	0.9300
O3—C8	1.251 (4)	C7—H7	0.9300
O3'—C8	1.240 (5)	C8—H8	0.9300
C1—C2	1.498 (3)	C8—H8'	0.9300
C2—C7	1.379 (3)		
			
O1W—Zn1—O1W^i^	91.11 (10)	C5—C4—H4	119.8
O1W—Zn1—O1^i^	100.59 (7)	C3—C4—H4	119.8
O1W^i^—Zn1—O1^i^	135.95 (7)	C4—C5—C6	120.0 (2)
O1W—Zn1—O1	135.95 (7)	C4—C5—C8	121.1 (2)
O1W^i^—Zn1—O1	100.59 (7)	C6—C5—C8	118.9 (2)
O1^i^—Zn1—O1	99.75 (9)	C7—C6—C5	119.6 (2)
Zn1—O1W—H1W1	117.8 (17)	C7—C6—H6	120.2
Zn1—O1W—H1W2	130.4 (17)	C5—C6—H6	120.2
H1W1—O1W—H1W2	111.8 (16)	C2—C7—C6	120.8 (2)
C1—O1—Zn1	102.62 (13)	C2—C7—H7	119.6
O2—C1—O1	120.65 (19)	C6—C7—H7	119.6
O2—C1—C2	121.9 (2)	O3—C8—O3'	116.3 (3)
O1—C1—C2	117.42 (19)	O3—C8—C5	123.1 (3)
C7—C2—C3	119.6 (2)	O3'—C8—C5	120.4 (3)
C7—C2—C1	120.14 (19)	O3—C8—H8	118.4
C3—C2—C1	120.2 (2)	C5—C8—H8	118.4
C4—C3—C2	119.5 (2)	O3'—C8—H8'	119.8
C4—C3—H3	120.2	C5—C8—H8'	119.8
C2—C3—H3	120.2	H8—C8—H8'	121.5
C5—C4—C3	120.4 (2)		
			
O1W—Zn1—O1—C1	−27.75 (17)	C3—C4—C5—C6	0.1 (4)
O1W^i^—Zn1—O1—C1	−130.56 (13)	C3—C4—C5—C8	−178.9 (2)
O1^i^—Zn1—O1—C1	88.73 (13)	C4—C5—C6—C7	−0.6 (4)
Zn1—O1—C1—O2	2.0 (2)	C8—C5—C6—C7	178.5 (2)
Zn1—O1—C1—C2	−175.83 (14)	C3—C2—C7—C6	−0.1 (3)
O2—C1—C2—C7	−17.2 (3)	C1—C2—C7—C6	−178.6 (2)
O1—C1—C2—C7	160.7 (2)	C5—C6—C7—C2	0.6 (4)
O2—C1—C2—C3	164.4 (2)	C4—C5—C8—O3	178.6 (3)
O1—C1—C2—C3	−17.8 (3)	C6—C5—C8—O3	−0.5 (4)
C7—C2—C3—C4	−0.3 (4)	C4—C5—C8—O3'	−7.0 (5)
C1—C2—C3—C4	178.2 (2)	C6—C5—C8—O3'	173.9 (3)
C2—C3—C4—C5	0.3 (4)		

Symmetry codes: (i) −*x*+1, *y*, −*z*+3/2.

### Hydrogen-bond geometry (Å, °)

**Table:**

*D*—H···*A*	*D*—H	H···*A*	*D*···*A*	*D*—H···*A*
O1W—H1W1···O1^ii^	0.84 (1)	1.93 (1)	2.761 (2)	174 (3)
O1W—H1W2···O2^iii^	0.84 (1)	1.88 (1)	2.720 (2)	174 (3)

Symmetry codes: (ii) −*x*+1, *y*−1, −*z*+3/2; (iii) −*x*+1, −*y*, −*z*+1.
